# Neural Stem Cells for Early Ischemic Stroke

**DOI:** 10.3390/ijms22147703

**Published:** 2021-07-19

**Authors:** Milton H. Hamblin, Jean-Pyo Lee

**Affiliations:** 1 Department of Pharmacology, Tulane University School of Medicine, 1430 Tulane Ave, New Orleans, LA 70112, USA; 2 Department of Physiology, Tulane University School of Medicine, 1430 Tulane Ave, New Orleans, LA 70112, USA; 3 Tulane Brain Institute, Tulane University, 1430 Tulane Ave, New Orleans, LA 70112, USA

**Keywords:** blood-brain barrier, matrix metalloproteinases, neural stem cells, stroke, tissue plasminogen activator, transplantation

## Abstract

Clinical treatments for ischemic stroke are limited. Neural stem cell (NSC) transplantation can be a promising therapy. Clinically, ischemia and subsequent reperfusion lead to extensive neurovascular injury that involves inflammation, disruption of the blood-brain barrier, and brain cell death. NSCs exhibit multiple potentially therapeutic actions against neurovascular injury. Currently, tissue plasminogen activator (tPA) is the only FDA-approved clot-dissolving agent. While tPA’s thrombolytic role within the vasculature is beneficial, tPA’s non-thrombolytic deleterious effects aggravates neurovascular injury, restricting the treatment time window (time-sensitive) and tPA eligibility. Thus, new strategies are needed to mitigate tPA’s detrimental effects and quickly mediate vascular repair after stroke. Up to date, clinical trials focus on the impact of stem cell therapy on neuro-restoration by delivering cells during the chronic stroke stage. Also, NSCs secrete factors that stimulate endogenous repair mechanisms for early-stage ischemic stroke. This review will present an integrated view of the preclinical perspectives of NSC transplantation as a promising treatment for neurovascular injury, with an emphasis on early-stage ischemic stroke. Further, this will highlight the impact of early sub-acute NSC delivery on improving short-term and long-term stroke outcomes.

## 1. Introduction

Stroke remains a leading cause of death and long-term disability in the United States, and ischemic stroke accounts for 87% of all strokes [1]. Aging is one of the primary risk factors for neurovascular diseases, and overall, two-thirds of strokes afflict patients over 65 years of age [2].

Clinically, ischemia-reperfusion (IR) leads to extensive neurovascular injury and neurological dysfunction [3,4]. Although there is a high incidence of ischemic stroke, treatment options are limited to mechanical endovascular treatment (thrombectomy) [5,6] and intravenous thrombolysis by tissue plasminogen activator (tPA) [7]. Moreover, major limitations of tPA treatment include a narrow therapeutic window within 4.5 h after stroke onset [8], and a greater potential for hemorrhagic transformation [9].

Strategies extending tPA’s and thrombectomy’s narrow time windows have been explored [10,11]. While thrombectomy has shown to be beneficial up to 24 h in a highly selected sub-group of patients by perfusion imaging [12,13,14,15], the outcome of tPA treatment between 4.5 h and 9 h post-stroke onset in selected patients with salvageable tissue is inconclusive [11]. However, delaying reperfusion might cause greater disruption of the blood-brain barrier (BBB), which is a promising target for reducing stroke injury. Preclinical studies show that IR triggers 2 episodes of BBB disruption: the first is reversible, but the second, which occurs following 24 to 72 h of ischemic stroke, is irreversible and contributes significantly to cell death [16]. Therefore, new strategies are needed to extend tPA’s therapeutic window, minimize deleterious effects, and improve stroke outcome.

Neural stem cells (NSCs) possess pleiotropic effects that are beneficial for early stroke pathophysiology, which is highly complex. Engrafted NSCs can differentiate into functional neurons in the brain [17], and also exhibit bystander (chaperone) effects that include delivery of neurotrophic factors, which could mitigate the toxic microenvironment and protect endangered host cells [18,19,20,21,22]. Preclinical studies demonstrate that engrafted NSCs improve stroke outcome through multiple mechanisms, such as protection of the BBB, decreased cerebral vascular inflammation, increased neurogenesis and angiogenesis, and enhanced neurological function [23,24]. Furthermore, therapeutic outcomes of NSCs can be different based on time and route of NSC administration. While current clinical trials focus on the outcome of stem cells on neuro-restoration by injecting cells during the chronic stroke stage [25], this review will focus on the therapeutic mechanisms and potential of NSC transplantation in the early (subacute) phase of ischemic stroke, thus improving long-term outcome.

## 2. Biology of Neural Stem Cells

### 2.1. Endogenous Neural Stem Cells

NSCs have functional properties of self-renewal and multipotency to generate neurons, astrocytes, and oligodendrocytes in the central nervous system (CNS). In mammals, NSCs are present in neurogenic “niches” [26] that include the subgranular zone (SGZ) in the dentate gyrus of the hippocampus [27] and the subventricular zone (SVZ) of the lateral ventricles [28,29,30]. While these areas are especially enriched in NSCs, experimental evidence suggests that neurogenesis can also occur in other brain regions such as the amygdala and the hypothalamus [31,32,33]. In the SVZ, along the walls of the lateral ventricles, progenitor cells proliferate, differentiate and migrate along the rostral migratory system [26]. With aging, there is a significant decrease in SVZ neurogenesis accompanied by changes in the niche, including diminished thickness [34], changes in the choroid plexus secretome [35], and increased microglial activation [36]. Furthermore, 3D image analysis reveals more significant vascular changes and NSC deficits in males, suggesting sex differences [37]. Intriguingly, several differences between the rodent and human SVZ have been reported [38]. NSCs are predominantly quiescent in healthy adults and neurogenesis is tightly regulated by the neurogenic niche [39,40]. Pathological insults such as ischemia stimulate neurogenesis in the brain [41,42,43]. However, injury-induced neurogenesis does not provide sufficient cells necessary for repairing extensive tissue damage after cerebral infarction [42,44]. Therefore, NSC transplantation could be a promising therapy to repair ischemia-induced neurovascular damage [19,45,46,47,48,49,50,51].

### 2.2. NSC Derivation

**Figure 1:**
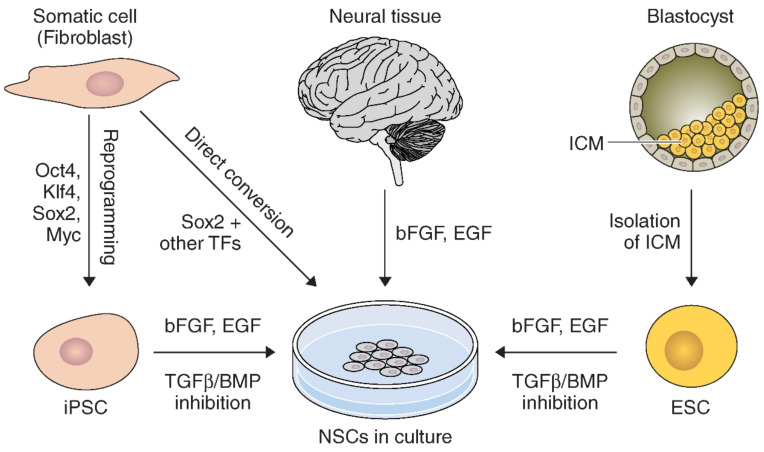
Schematic diagram of NSC derivation through diverse methods and sources. NSCs can be directly extracted from neural tissue and expanded in vitro. Also, NSCs can be reprogrammed from iPSCs or ESCs by specific combinations of differentiation factors. Further, NSCs can be generated by direct conversion of somatic cells omitting the iPSC derivation step. ICM, inner cell mass.

Neural stem cells can be derived by many approaches from various sources (Figure 1). NSCs can be directly harvested from neuroectoderm in fetal tissue, or the SVZ and SGZ in adults [52]. These primary NSCs can be expanded and maintained in culture using basic fibroblast growth factor (bFGF) and epidermal growth factor (EGF) [52].

NSCs can also be derived from other sources. For example, NSCs can be generated from embryonic stem cells (ESCs) [53,54,55]. However, a great deal of manipulation and intermediate steps are required for ESCs to fully differentiate into NSCs [53,55]. Neuroinduction of ESCs in culture can be achieved by inhibiting TGFβ/BMP signaling pathways during expansion with bFGF and EGF [56].

Similarly, NSCs can be generated from induced pluripotent stem cells (iPSCs) [56,57]. Many types of somatic cells can be readily obtained to dedifferentiate into iPSCs. These include fibroblasts, keratinocytes [58], blood [59], and hair follicles [60]. Notably, a similar methodology using dual-inhibiting SMAD signaling for NSC derivation from ESCs can generate iPSCs into NSCs [56]. However, generating iPSCs requires extra reprogramming to dedifferentiate somatic cells [61]. Microarray analysis studies confirmed that iPSC-NSCs and ESC-NSCs have very similar but not identical genetic expression profiles [62,63]. Use of iPSCs is advantageous due to fewer ethical concerns and pathological immune responses, since they can be generated by reprogramming from the patient’s own tissue [57]. iPSC-NSCs have been reported to be therapeutic in preclinical neurological disease models, which raises hope as a clinically promising source for cell therapy.

Direct conversion methods for reprograming somatic cells into induced NSCs (iNSCs) have been developed [64,65]. For instance, mouse fibroblasts were successfully transformed into NSCs by the introduction of pluripotency factors Oct4, Sox2, Klf4, and c-Myc [64]. Similarly, NSCs can be generated by constitutively inducing Sox2, Klf4, and c-Myc while stringently limiting Oct4 activity to the initial phase of reprogramming [65]. Generation of iNSCs has been reported with different combinations of NSC-specific transcription factors as well [66,67]. Further, iNSCs can also be generated by a single transcription factor, *Sox2* or *ZFP521* from mouse and human fibroblasts [68,69]. Pharmacological reprogramming can be used to generate iNSCs by signaling-directed transcriptional activation [70]. A direct iNSC generation methodology provides a new strategy for the generation of NSCs through direct cell transdifferentiation and avoids the lengthy intermediate step to generate iPSCs. Thus, direct conversion of somatic cells to NSCs can be a more efficient strategy [65]. Further, iNSCs from the patient’s own cells lower the risk of rejection of iNSCs following transplantation [71]. Therefore, research advancements in direct differentiation of NSCs can be promising for future therapeutic applications against stroke.

### 2.3. Labeling and Tracking Exogenous NSCs

Various methods for labeling and tracing NSCs have been established. Direct labeling is a widely used method by introducing a marker into stem cells or onto the cell surface before transplantation. Prelabeling NSCs with the thymidine analog, bromodeoxyuridine (BrdU), which incorporates into the nuclei during the S phase, is a reliable method. Newly-divided BrdU^+^ NSCs can then be detected with an antibody [52]. This method is useful to quantify the number of divisions of engrafted NSCs following transplantation [52] and is preferentially used for short-term tracking studies because BrdU becomes diluted over time [52]. To trace engrafted NSCs using clinically translational magnetic resonance imaging (MRI), NSCs can be labeled with super-paramagnetic iron oxide particles (SPIO) [72]. Stem cells can also be effectively labeled with fluorescent probes (e.g., orange cell tracker CMTMR [73] or CM-Dil [74]) for optical imaging and also radiotracers for radionuclide imaging [75]. However, direct radiotracer labeling can lead to potential radiation damage to the cells.

NSCs can be labeled via viral transduction to express readily identifiable markers such as GFP and lacZ. However, this method raises concerns about transgene inactivation and altering the properties of stem cells [52]. When human NSCs (hNSCs) are engrafted into rodents (e.g., species mismatch), hNSCs can be identified by human-specific antibodies. Engrafted stem cells can also be verified by donor-to-recipient sex-mismatch [52].

### 2.4. Stem Cell Migration

NSCs can migrate to areas of injury and neurodegeneration in the CNS. Migration and engagement of NSCs with a pathologic niche is the first step in cell-mediated restoration of homeostasis to the injured region [18,76,77,78,79]. NSC pathotropism, which is the natural tendency of homing to the site of injury, is partly achieved by chemokine receptors on NSCs that respond to proinflammatory cytokines secreted from the pathologic region [80,81,82,83,84,85,86]. For example, chemokine stromal cell-derived factor-1α (SDF-1α/CXCL12) interacts with CXCR4 receptors on NSCs as a major step in directing NSCs to injured brain regions [74,76,83,87]. The role of SDF-1α in stem cell homing is also implicated in cardiovascular and renal disease [88,89,90]. Further, in vitro transwell chemotaxis experiments demonstrated that NSCs preferentially migrate toward a higher SDF-1α concentration [76,91] and also synthetic SDF-1α [76]. SDF-1α is increased in stroke-affected brain tissue, and NSCs migrated toward the infarct area [92]. Targeting the SDF-1α/CRCX4 signaling pathway could be a highly efficient strategy for increasing the migration capacity and number of NSCs that cover the stroke-afflicted site.

Stroke injury generates an irreversible “necrotic core” and also a salvageable “penumbra” that surrounds the core. The penumbral region is metabolically active and structurally intact for longer than the infarct core. Intracranial transplantation of NSCs into the penumbral parenchyma has been reported to be beneficial [52,78,79,93]. Also, migration of NSCs from the intravascular space to the pathologic niche has been shown after tail vein [94] or intra-arterial [95] injection. However, intravenous delivery of stem cells can be trapped in filtering organs including the lung and liver [96,97,98,99]. Thus, intra-arterial injection may provide a more direct route to the lesion and exhibit better stroke outcome [46,100]. Also, intranasally delivered stem cells have been reported to extensively migrate to areas afflicted by experimental hypoxia-ischemia and ischemic stroke [101,102].

## 3. Pathophysiology of Early Ischemic Stroke

### 3.1. Blood-Brain Barrier and Ischemia-Reperfusion Injury

Integrity of the BBB is very critical as it forms a physical barrier created by tight junction proteins between endothelial cells that strictly regulate transcellular trafficking [103,104,105,106]. The BBB is created by spatiotemporal coordination between neurons, astrocytes, vascular cells (e.g., endothelial cells and pericytes), and the extracellular matrix (ECM). This neurovascular unit (NVU) is crucial for maintaining CNS homeostasis [107,108,109]. The BBB also controls leukocyte trafficking into the CNS for immune surveillance and response to infections [110], or removal of tissue damage [111].

BBB dysfunction is a prominent pathophysiological feature of acute ischemic stroke. Acute ischemia results in rapid decrease of cerebral blood flow and causes metabolic dysregulation due to oxygen and glucose deprivation. Consequent decrease of energy supply leads to lactic acidosis, alterations in ion transport, and excess extracellular accumulation of glutamate [112], which results in endothelial swelling and BBB damage [113].

Restoration of cerebral blood flow supplying oxygen and nutrients is crucial to attenuating ischemic stroke damage. However, reperfusion following ischemia further challenges the BBB, which causes a biphasic opening of the BBB. Experimental studies report that the initial phase of the BBB opening is reversible and occurs within several hours after reperfusion [114], but the second opening is irreversible and occurs 24–72 h after reperfusion [115]. While the exact mechanisms underlying increased BBB permeability in the early IR stages remain under extensive investigation, accumulating evidence shows that IR promotes greater disruption of the BBB through reactive oxygen species (ROS) damage to cellular molecules, upregulation of inflammatory factors and matrix metalloproteinases (MMPs), and alterations of tight junction proteins (TJPs) [116,117,118]. The initial BBB opening is linked to subtle alterations of tight junction complexes [119,120], followed by a second opening involving enzymatic cleavage of TJPs [114,121,122].

### 3.2. MMPs

MMPs are a family of zinc-binding proteolytic enzymes that are capable of degrading components of the ECM [123]. Although MMPs play critical roles in angiogenesis, tissue remodeling, and wound healing [124,125], there is a growing body of research highlighting strong associations between unrestrained MMP activity, neuroinflammation, and progression of neurodegenerative disease [126,127,128,129,130,131]. MMPs play a dual role following ischemic stroke [132], enhancing neurovascular injury during the acute phase but playing a beneficial role during the chronic recovery state. Neuroinflammation-associated MMPs degrade perivascular basement membranes and TJPs of the BBB, which contribute to increased BBB leakiness and the passage of toxic substances into ischemic tissue [133]. Consequences of damage to tight junctions in the BBB include upregulation of proinflammatory cytokines and infiltration of peripheral immune cells into the CNS.

#### 3.2.1. MMP-2 and MMP-9

Already, the involvement of MMP-2 and MMP-9 has been extensively studied in stroke. Following stroke, the levels of MMP-2 and MMP-9 are elevated in ischemic tissue [134,135] and they contribute to BBB disruption [136,137]. For example, prior studies show upregulation of MMP-2 in the acute stages of IR in rodents and nonhuman primates [138,139]. Also, during the first 24 h post-IR, there is a significant increase in the level of MMP-9, which is associated with more extensive damage to TJPs, especially zona occludens-1 (ZO-1), a protein that connects the actin cytoskeleton of microvascular endothelial cells (ECs) to occludin [137,140]. Further, previous studies have reported that MMP-9 activity is positively correlated with BBB breakdown after stroke [141] and linked to tPA-induced hemorrhage in stroke patients [142] and animal models [143,144]. TJPs, such as claudin-5, occludin, and ZO-1 are crucial for BBB integrity [120,145]. It has been shown that at least one of the mechanisms by which MMP-9 participates in BBB damage is through the degradation of specific TJPs [146]. Preclinical investigations using the well-established experimental stroke model, middle cerebral artery occlusion-reperfusion (MCAO/R), report that BBB leakage begins to occur 24 h post-MCAO/R, regardless of claudin-5 or occludin disruption [147]. However, MCAO/R does result in the destruction of ZO-1 [145]. Given that MMP-9 is known to degrade ZO-1 [137,148], MMP-9 inhibition may ameliorate proteolytic degradation of ZO-1 and preserve BBB integrity.

#### 3.2.2. MMP-3 (Stromelysin-1)

MMP-3, a 51-kDa protein [149], is one of the major inducible MMPs that can activate latent pro-MMP-9 [150,151,152]. MMP-3 has broad substrate specificity and can degrade various ECM proteins including fibronectin, denatured collagens (gelatin), laminin, and proteoglycans [153], and is critical to brain tissue remodeling and wound healing. Injury-induced MMP-3 is upregulated within several hours after stroke, suggesting association with the initial opening of the BBB [154]. MMP-3 deficient mice exhibited reduced degradation of TJPs of the BBB and less neutrophil infiltration caused by intracerebral lipopolysaccharide (LPS)-induced BBB opening [128] or spinal cord injury [130] in young adult mice. Further, MMP-3 reportedly exacerbates tPA-induced intracerebral hemorrhage (ICH) post-stroke in thrombotic MCAO mouse brains [154]. While knockout of MMP-3 in stroke mice reduced the tPA-enhanced risk of ICH, delayed tPA administration (4 h post-MCAO) in the thrombotic ischemic stroke model was found to further augment MMP-3 expression selectively in ECs in the ischemic hemisphere, which suggests the involvement of MMP-3 in disruption of the BBB and ICH. While MMP-9 expression is also significantly upregulated in wild-type mouse ischemic brains, ICH was less prominent in MMP-9 KO mice. However, the location and levels of MMP-9 were not altered following tPA treatment 4 h post-MCAO, suggesting that MMP-3 may be a greater contributor to tPA-induced ICH than MMP-9 [154]. Combined laser microdissection and protein array studies showed significant upregulation of MMP-3 in the human ischemic stroke brain, along with MMP-9 [155].

In vitro, MMP-3 is upregulated either by tPA treatment or ischemic conditions in cultured murine brain ECs. However, this effect is attenuated by inhibition of either lipoprotein receptor-related protein (LRP), a scavenger receptor that can bind tPA [156], or nuclear factor-kappa B (NF-κB) activation, suggesting an underlying mechanism of MMP-3 induction via the LRP/NF-κB pathway [157]. While the role of MMP-3 in hemorrhagic transformation in rodents has been reported [154,158,159], the critical role of MMP-3 in BBB integrity and function remains understudied in cerebral IR injury post-stroke.

### 3.3. Inflammatory and Immune Responses after Stroke

Inflammation is a prominent feature of early stroke pathophysiology [160,161]. Stroke leads to increased BBB permeability that enables infiltration of neutrophils, macrophages, and T lymphocytes into the CNS [162]. Although inflammation is crucial for brain repair, an unchecked inflammatory response is detrimental in early ischemic stroke [163].

Microglia and macrophages play critical roles in modulating CNS repair [164]. Cerebral ischemic injury activates resident microglia that constantly survey their surroundings in the brain. Microglia exist in two functional phenotypic states and there is a dynamic interplay between their functional status responding to extracellular signals. For example, ischemic stroke promotes M1 microglial polarization, which leads to secretion of detrimental factors such as TNF-α, IL-1β, and ROS [165]. Alternatively, activation of M2 microglial polarization promotes the resolution of neuroinflammation by releasing neurotrophic factors and anti-inflammatory cytokines such as IL-10 and TGF-β [165,166].

Less than 24 h after stroke, peripheral immune cells start to infiltrate the brain through the compromised BBB [167]. Macrophages, the major inflammatory cell infiltrate during acute stroke [168], are highly plastic and can display functionally different phenotypes [169]. M1 macrophages increase CNS damage by secreting proinflammatory molecules including TNF-α, IL-8, and IL-12 [161,170]. Infiltrating M1 macrophages recruit neutrophils into the CNS through secretion of IL-8, which promote further inflammation and tissue damage by releasing NO, MMPs, and cathepsins [161]. Secreted factors by both macrophages and neutrophils constitute the initial inflammatory cascade after stroke. In contrast, inflammation-resolving M2 macrophages release anti-inflammatory cytokines such as TFG-β and IL-10 [161,171]. Further, M2 macrophages are also beneficial for stroke outcome by removing ischemic debris [170].

Neuroinflammation after stroke is eventually resolved and the M1-to-M2 shift occurs when proinflammatory mediators become further reduced by anti-inflammatory mediators [161]. The involvement of the STAT family is reported in regulating functional status of immune cells [172,173]. While STAT6 signaling drives M2 macrophage polarization [173,174], STAT1 activation promotes M1 macrophages [175]. IL-4, a STAT6 activator, drives microglia and macrophages toward a beneficial M2 phenotype and facilitates stroke recovery [176].

After macrophage and neutrophil infiltration, CD4^+^ and CD8^+^ T cells enter the CNS through the damaged BBB [177,178]. CNS-specific Th1 cells secrete interferon gamma (IFNγ) after stroke [179] and activate cytotoxic CD8^+^ T cells that exacerbate CNS damage [180].

Efferocytosis, phagocytic clearance of dying or dead cells by microglia and macrophage infiltrates [161,181], contributes to resolving inflammation and restoring brain homeostasis post-stroke.

### 3.4. Ischemic Tissue Loss and Neurological Dysfunction

Stroke results in extensive cell death and tissue infarction in the affected region. The brain consumes high levels of oxygen and glucose and relies on oxidative phosphorylation for its energy source. Ischemia limits oxygen and glucose supplies thus, leading to significant loss of endogenous energy stores and disruption of ionic balance or neurotransmitter reuptake [182]. In particular, a metabolic insult after stroke leads to extracellular accumulation of glutamate, which activates NMDA and AMPA receptors, leading to high calcium influx and consequent cell death [183]. For example, Ca^2+^ overload drives mitochondrial function toward releasing cytochrome C, which activates cell death signaling pathways [184,185].

Edema is also major contributor of cell death and tissue infarction after ischemic stroke. Hyperactivation of glutamate receptors leads to increased Na^+^ and water influx, which causes hypotonic-induced cell swelling [183]. Furthermore, BBB damage enables the entry of foreign molecules, fluid, and immune cells into brain interstitial spaces, which promotes vasoactive edema and exacerbates tissue injury [186]. Although vasoactive edema is eventually resolved, clearance of debris and angiogenesis are often too slow to attenuate acute edema-linked brain injury after stroke.

Neuronal circuitry is also disrupted after stroke. Excitotoxicity and persistent depression of inhibitory neurotransmission by excess extracellular glutamate [183,187] and reduced GABA_A_ receptor expression [188], respectively, reduces neurological outcome after stoke.

## 4. Endogenous Repair Mechanisms

### 4.1. Angiogenesis

Spontaneous stroke recovery occurs [189,190] despite the fact the adult brain has limited endogenous repair capabilities [191]. Angiogenesis, the formation of new capillary blood vessels from preexisting vessels, occurs as a result of cerebral ischemia and can contribute to CNS plasticity and post-stroke recovery [192,193,194,195,196]. Endothelial cell proliferation occurs in the subacute stage of stroke and can continue for several weeks due to increased expression of angiogenesis genes and growth factors [197,198,199]. While angiogenesis can improve the repair process following stroke, the initial angiogenic vascular remodeling process is accompanied with a compromised endothelial barrier function. For example, vascular endothelial growth factor (VEGF) is the major initiator for stimulating angiogenesis and is highly upregulated as early as 1 h following cerebral ischemia. However, VEGF also promotes vascular permeability and barrier dysfunction [200]. Therefore, VEGF delivery aiming for increasing angiogenesis in early phase stroke can be both beneficial and detrimental. For instance, targeted local delivery in the subacute stage is better tolerated [201] but intraperitoneal systemic injection of VEGF in the early phase of stroke exacerbated BBB leakage, vasogenic edema and further increased infarct volume. Moreover, studies focusing on VEGF-induced BBB leakage demonstrated that blocking VEGF receptors after ischemia mitigates BBB permeability, brain edema, and reduces cerebral infarct volume [201]. Furthermore, regarding increased BBB permeability for initial stages of angiogenesis, high levels of MMPs such as MMP-2 and MMP-9 [202] has been associated with ECM remodeling during angiogenesis.

An angiogenic response is stimulated in the potentially salvageable penumbra for post-stroke recovery [192]. A higher vessel density in the penumbra is linked to improved survival after ischemic stroke [192,199,203], and further penumbra damage following stroke can have a catastrophic neurological outcome [204]. Angiogenic growth factors play an important role for cell survival in the penumbra [205]. In addition to enhancing local blood supply after stroke, angiogenesis also promotes the removal of necrotic brain tissue [206,207]. However, angiogenesis can be negatively influenced by several factors, including aging [208]. Given that stroke usually affects the aging population and clinical trials using stem cells show beneficial outcome in stroke patients [47], translational studies investigating the effects of neural stem cells on vascular remodeling during stroke in aged animals is greatly warranted.

### 4.2. Endogenous Neurogenesis

Endogenous neurogenesis is activated following stroke [42,44,49]. NSCs proliferate, generate new neurons and secrete trophic factors [41,42,50,189]. Further, neurogenesis and angiogenesis are coupled in the neurovascular unit in that endothelial cells release trophic factors that regulate both responses [209,210], and VEGF as a possible mediator for coupling angiogenesis and neurogenesis after stroke [210]. Microvessel characteristics also change after stroke [211], and neuroblasts migrate to the vascular remodeling site [212]. Additionally, NSCs enhance angiogenesis through trophic factors and can improve cerebral capillary blood flow [210,213]. These studies highlight stroke-induced coupling between neurogenesis and brain vascular remodeling. However, enhanced endogenous neurogenesis is not sufficient to yield substantial impact on repairing neuronal damage [42,214,215]. Therefore, transplantation of exogenous NSCs may be an effective strategy to reduce stroke brain damage.

Astrocytes also play a critical role in neurogenesis and angiogenesis by secreting neurotrophic and vascular growth factors [216,217]. Astrocytes can also mediate neurotransmission and help maintain neurovascular coupling [218]. During ischemia, astrocytes show neuroprotective effects including the removal of excess glutamate and enhancing revascularization [219,220,221]. However, in the chronic stroke phase, astrocytes can diminish neurological recovery by releasing growth inhibitory factors and forming glial scars, which lead to poor neuronal connectivity [222]. Thus, early stroke intervention with stem cells may reduce chronic stage stroke complications.

## 5. Transplantation of Pleiotropic Neural Stem Cells for Ischemic Stroke

There are prominent changes in the brain during initial ischemic insult. The timing of NSC transplantation is critical to the repair and regenerative therapeutic actions for ameliorating the complex, multifactorial pathophysiology of ischemic stroke. Engrafted NSCs release therapeutic trophic factors for brain tissue remodeling and facilitating neuronal plasticity [79,223,224]. NSC-secreted brain-derived neurotrophic factor (BDNF) [77,79] is a main neurotrophin that promotes neuroprotection, neurogenesis, and enhances neurological outcome following stroke [225,226,227,228]. Also, neurotrophic factors (NTFs) assist in maintaining neuronal health, ECM remodeling, and cell proliferation. NTFs also protect neuronal tissue from extensive damage [229]. VEGF is an important neurotrophic factor that facilitates angiogenesis and is involved in neural tissue repair [205,230]. Further, VEGF regulates mitogenesis and survival for vascular endothelial cells [231,232] and provides neuroprotective effects against ischemic injury [205,233,234]. When VEGF is pharmacologically inhibited using SU1498 and Flt-1Fc, NSC-mediated protection is dramatically thwarted [235]. Additionally, other neurotrophic factors such as ciliary neurotrophic factor, glial cell-line derived neurotrophic factor, and neural growth factor contribute to neuroprotection and repair [236,237,238,239,240,241]. In addition to secreting NTFs, engrafted NSCs generate electrophysiologically active functional neurons that make appropriate synaptic connections with host neurons [242].

### 5.1. Transplantation of NSCs for Early Stroke Intervention

Mostly, pharmacological-based therapy has been implemented to reduce acute/sub-acute stroke injury. For instance, minocycline, a commonly used semi-synthetic tetracycline antibiotic, has demonstrated potential as a safe, efficacious neuroprotectant for clinical treatment of stroke [243,244], partly attributed to reducing inflammation, cell death, and BBB damage [245,246,247,248,249,250]. However, NSC administration at the subacute stage may be used as part of a combinatorial strategy to protect neurons by reducing brain injury and cell death post-stroke. Preclinical studies report that NSC transplantation 24 h post-stroke leads to reduced inflammation and BBB damage, and better functional stroke outcome in mice [51,77,78,79]. The beneficial actions of NSC transplantation during early stroke are highlighted below.

#### 5.1.1. BBB Support and MMPs

The BBB has great functional significance for protecting against the entry of neurotoxic agents and inflammatory mediators. Thus, preserving BBB integrity and function is a promising strategy for ischemic stroke. Early NSC delivery ameliorates BBB injury post-stroke. Intracranial hNSC administration in mice 24 h post-stroke leads to robust NSC migration to the injured region, decreased infarct size, and lessened BBB disruption [79]. This rapid therapeutic response of NSCs clearly suggests anti-inflammatory mechanisms of action. Consistent with these findings, hNSC-engrafted mice showed decreased microglia activation and reduced expression of IL-6, IL-1β, and MIP-1α [79]. Also, intracranial administration of hiPSC-NSCs in mice 24 h following stroke leads to decreased leakage of IgG into the brain parenchyma [78].

During ischemia reperfusion, MMPs are significantly upregulated in affected tissue and degrade the ECM. Higher MMP-9 activity is correlated with BBB disruption during stroke [148,251] and dysfunction of BBB tight junctions [78]. However, MMP-9 activity after stroke was decreased in NSC-transplanted young adult [78,79] and aged mice [77]. For instance, Western blot analysis shows that transplanted hNSCs attenuated MMP-9 at 48 h post-stroke, suggesting that hNSCs can reduce BBB disruption [79]. This finding was validated by gel zymography that measures MMP-9 enzyme activity. While MMP-9 activity is upregulated at 48 h post-stroke, engrafted hNSCs significantly reduced MMP-9 activity. In contrast, MMP-2 was low 48 h post-MCAO/R, implying that MMP-2 may not be a significant contributor at this time point. MMP-9 induction is associated with tPA-induced hemorrhage in stroke patients [142] and animal models [143,144]. MMP-9 is further elevated by delayed tPA treatment and associated with exacerbated BBB breakdown during stroke [141,148,251]. Aged mouse stroke brains show that hNSC administration reduces MMP-9 following delayed tPA treatment [77], suggesting BBB protection.

Tight junction proteins are essential in modulating BBB integrity [120,145]. However, cerebral ischemia leads to destruction of the tight junction protein, ZO-1 [145]. MMP-9 is known to degrade ZO-1 [137,148] thus hNSC-mediated MMP-9 downregulation may reduce ZO-1 degradation and consequently preserve BBB integrity. Indeed, hNSC-transplantation in mice showed higher ZO-1 [77], suggesting hNSCs reduced degradation of ZO-1. Since delayed tPA is known to augment the severity of neural cell death and BBB damage, adjuvant therapy with hNSCs after tPA administration may be an effective strategy to not only retain thrombolysis for acute ischemic stroke but also reduce BBB disruption in the early stage of stroke.

#### 5.1.2. Inflammation

**Figure 2:**
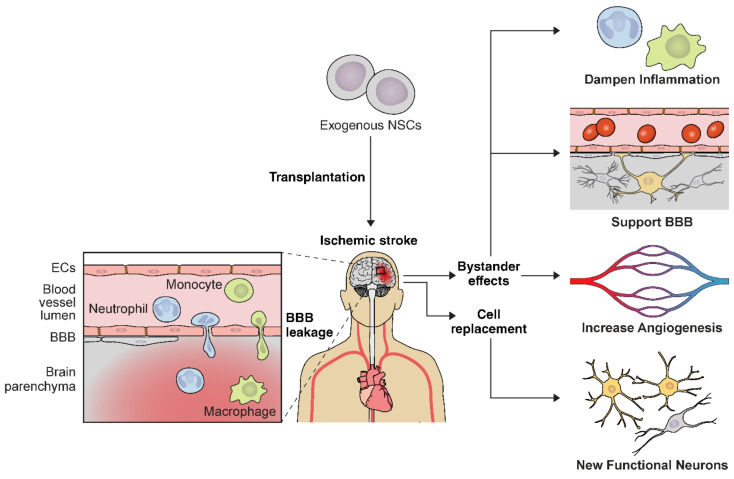
Schematic diagram of NSC delivery illustrating multiple therapeutic mechanisms in ischemic stroke. NSCs can differentiate into functional neurons in the stroke brain and possess pleotropic bystander effects. These bystander effects include attenuation of blood-brain barrier (BBB) disruption, increased angiogenesis, and modulation of immune responses after ischemic stroke. ECs, endothelial cells.

Ischemic stroke activates astrocytes and microglia and leads to inflammatory cell infiltration in the affected tissue due to increased BBB permeability. NSC delivery in the early stroke phase attenuates this complex inflammatory signaling cascade [223,252,253] (Figure 2). NSCs ameliorate ischemic damage by lowering proinflammatory mediators including TNF-α, IL-1β, IL-6, MCP-1, and Iba-1 [223,253]. For instance, hiPSC-NSC transplantation is associated with significantly decreased Iba-1 positive cells in the stroke mouse model [254]. Transplanted mice also had decreased numbers of GFAP-positive astrocytes [254]. Similarly, NSC-engrafted mice displayed lower CD45^+^ and Iba-1^+^/major histocompatibility II immune cells after ischemia [253]. Collectively, these studies demonstrate an important role of NSCs in BBB integrity and functional recovery from cerebral ischemia through influencing the extracellular microenvironment and reducing neuroinflammation. Thus, implementing stem cell therapies for immunomodulation and targeting the proinflammatory signaling cascades in the brain may be an effective therapeutic strategy for early ischemic stroke.

### 5.2. Long-Term Outcome of NSC Transplantation at the Early Stroke Phase

NSC transplantation in the early acute/subacute phases of stroke aims for neuroprotection from the deleterious extracellular surroundings after ischemia. Further, NSC delivery during the subacute phase will also stimulate neurorestorative mechanisms to benefit long-term outcome.

#### 5.2.1. Angiogenesis

NSC transplantation promotes stroke recovery by stimulating angiogenesis, which is coupled to neurogenesis [209]. Early hNSC delivery post-stroke enhanced angiogenesis in rats [255]. When assessed at 14d after NSC delivery, BrdU^+^/vWF^+^ proliferating endothelial cells were increased in the afflicted area of NSC-engrafted rats, demonstrating increased angiogenesis [255]. Also, transplantation of NSCs overexpressing Cu/Zn-superoxide dismutase (SOD1) into peri-infarct mouse cortex 2d post-stroke enhanced angiogenesis, which is potentially mediated by upregulation of VEGF [256].

#### 5.2.2. Cell Replacement

Early delivery of NSCs may facilitate stroke recovery through generation of new neurons or enhancing the neurogenic response. For instance, engrafted hNSCs into the rat striatum 2d post-stroke differentiated into neurons at 6 weeks and enhanced endogenous neurogenesis [257]. Similarly, other studies reported direct neural replacement after early NSC delivery during stroke. Engrafted hNSCs gave rise to neurons and astrocytes in the rat brain when they were implanted one day post-stroke [258,259]. When assessed at 30d post-transplant, the majority of engrafted iPSC-NSCs at 24 h post-stroke remained as undifferentiated NSCs, and only a modest amount of hiPSC-NSCs were positive for the neuronal marker (TuJ-1) [78]. Based on these findings, direct neural replacement may not be a primary reason for the beneficial effects of early NSC administration post-stroke.

#### 5.2.3. Improved Neurological Outcome

Improved neurological behavior is considered to be the gold standard for evaluating long-term stroke outcome. Modified Neurologic Severity Score (mNSS) tests revealed improved neurological function two weeks post-transplant when hNSCs were engrafted at 24 h in rat stroke brains [255]. Similarly, intraventricular transplantation of rat NSCs overexpressing HIF-1α [260] or intravenous injection of mouse NSCs overexpressing bFGF [261] into rat brains 24 h after MCAO promoted behavioral recovery as assessed over 4 weeks by NSS scoring. Also, neurological function rapidly improved by 2d post-stroke when mice were engrafted with hNSCs 24 h post-MCAO [79]. These hNSC-engrafted mice displayed improved sensory motor function when assessed by the forepaw adhesive removal test [262]. In addition, engrafted mice show improved balance and motor coordination as evaluated by the beam walk and rotarod tests. Improved neurological outcome persisted when assessed for a month [79], suggesting that early stroke intervention with NSCs can lead to improved long-term neurological outcomes in ischemic stroke patients. Other studies also reported that intracranial iPSC-NSC delivery 24 h post-stroke leads to improved long-term neurological function [78]. Moreover, NSCs engrafted into the hippocampus expedited rapid migration of NSCs to injury sites and enhanced stroke recovery [78,79]. Another study using the murine NSC cell line, MHP36, showed greater functional recovery after intracranial injection 2d post-stroke in mice [263]. hNSC-engrafted animals showed better behavioral function when monitored for 28d post-stroke [263]. Furthermore, transplantation of hNSCs overexpressing *Bcl2l1* into the infarct site 2d after permanent MCAO showed improved neurological function in mice [264]. Also, intracranial administration of SOD1-overexpressing NSCs 2d post-stroke improved behavioral performance as assessed over 28d by mNSS scoring and the rotarod test [256]. Based on these findings, early intervention of stroke with NSC administration clearly shows significant improvement for long-term neurological outcome and behavioral function.

## 6. Conclusions

To address the complex pathophysiology of neurovascular diseases, therapeutic strategies include pharmacologic, genetic, and cell-based tissue engineering. NSCs can be used alone or in combination with other interventions that can work synergistically. Specifically, NSCs possess anti-inflammatory actions that attenuate delayed tPA-associated adverse effects of early stroke. Further, diabetes increases the risk for stroke and worsens overall stroke outcome [265,266]. Diabetic patients are not eligible for tPA treatment due to greater risk of BBB permeability and hemorrhagic transformation caused by complications of ischemic stroke [265]. Diabetic db/db stroke mice show a further increase in the MMP-9 mRNA level and activity and inflammation [267]. Therefore, new therapeutic strategies to treat stroke patients with comorbidities are needed. Stem cell-based therapy for human trials is currently focusing on enhancing stroke rehabilitation by engrafting stem cells during the stroke recovery phase [47,268,269]. Phase 1 and 2 clinical trials delivering human NSCs during the chronic stroke phase were reported to show no safety concerns and improve motor recovery [268,270]. However, stem cell delivery during the sub-acute stroke phase may benefit more patients by ameliorating early stroke injury and consequently improving later stroke outcome. Therefore, deeper insight into early-phase stroke injury and identifying optimal stem cell strategies are needed for successful translation.

## Data Availability

Data sharing not applicable to this article as no datasets were generated or analyzed during the current study.

## Abbreviations

**Abbreviation**	**Description**
AMPA	α-Amino-3-hydroxy-5-methyl-4-isoxazolepropionic acid
BBB	Blood-brain barrier
BDNF	Brain-derived neurotrophic factor
CNS	Central nervous system
CXCR4	C-X-C chemokine type 4 receptor
ECASS-4	European Cooperative Acute Stroke Study-4
ECM	Extracellular matrix
EGF	Endothelial growth factor
ESC	Embryonic stem cell
FGF	Fibroblast growth factor
GFP	Green fluorescent protein
ICH	Intracerebral hemorrhage
IL-1β	Interleukin-1β
iPSC	Induced pluripotent stem cell
IR	Ischemic reperfusion
LRP	Lipoprotein receptor-related protein
MCAO	Middle cerebral artery occlusion
MCP-1	Monocyte chemoattractant protein-1
MIP-1α	Macrophage inflammatory proteins
MMP	Matrix metalloproteinase
NF-κB	Nuclear factor kappa B
NMDA	N-Methyl-D-aspartic acid or N-methyl-D-aspartate
NSC	Neural stem cell
NTF	Neurotrophic factor
NVU	Neurovascular unit
ROS	Reactive oxygen species
SDF-1α	Stromal cell derived factor 1-α
STAT	Signal transducer and activator of transcription
SVZ	Subventricular zone
TIMP-2	Tissue inhibitor of metalloproteinases 2
TJPs	Tight junction proteins
TNF-α	Tumor necrosis factor
tPA	Tissue plasminogen activator
VEGF	Vascular endothelial growth factor
vWF	von Willebrand factor
ZO-1	Zonula occludens-1 (ZO-1)
